# Editorial: Redox Metabolism in Environmental and Ecological Physiology of Animals

**DOI:** 10.3389/fphys.2022.904746

**Published:** 2022-04-25

**Authors:** Daniel C. Moreira, Youji Wang, Giancarlo López-Martínez, Marcelo Hermes-Lima

**Affiliations:** ^1^ Research Center on Morphology and Applied Immunology, Faculty of Medicine, University of Brasília, Brasília, Brazil; ^2^ International Research Center for Marine Biosciences at Shanghai Ocean University, Ministry of Science and Technology, Shanghai, China; ^3^ Department of Biological Sciences, North Dakota State University, Fargo, ND, United States; ^4^ Department of Cell Biology, Institute of Biological Sciences, University of Brasília, Brasília, Brazil

**Keywords:** antioxidant, dehydration, estivation, oxidative stress, radiation, reactive oxygen species, reproduction

The accumulation of O_2_ in atmosphere and water bodies over hundreds of millions of years was accompanied by the evolution of life (Planavsky and Mills, 2021), which includes the development of an intricate cell signaling system composed of antioxidants, reactive oxygen species (ROS) and target proteins (e.g., those containing thiolate groups, methionine, metal centers, selenocysteine) (Sies et al., 2017). Recent research has provided increasing evidence that reactive sulfur species (RSS) played a key role in this process (Olson, 2019; Olson, 2020). Reactive oxygen species, which are inevitable products of aerobic life, were considered only harmful biochemicals until the early 2000’s. Since then, ROS have been viewed as key cell messengers, essential for all oxygen-dependent life forms (Sies and Jones, 2020). In animals, the generation of ROS by multiple pathways (e.g., mitochondrial respiratory chain, NADPH oxidases and other enzymes) and their management by stress responsive pathways (e.g., endogenous antioxidants, transcription factor, chaperones and repair systems) compose the redox metabolism (Hermes-Lima, 2004; Jones and Sies, 2015). The interaction of reactive species, not only oxygen-derived (ROS) but also nitrogen- (RNS) and sulfur-derived (RSS), among themselves and other molecular targets makes up the reactive species interactome (Cortese-Krott et al., 2017). This plays a key role in the maintenance of homeostasis and responds to external stimuli accordingly (Sies, 2020). In the last few years, our understanding of the interplay between the redox metabolism and animal ecophysiology has drastically advanced. Changes in redox metabolism have been documented for phylogenetically diverse species exposed to a myriad of environmental stressors, including natural (e.g., hypoxia, droughts, and UV radiation) and anthropogenic (e.g., chemical contaminants) sources. Several of these abiotic factors (e.g., water availability, oxygen availability, and radiation incidence) fluctuate within variable time frames (e.g., daily, seasonally) and ultimately affect the fitness of organisms. In “*Redox Metabolism in Environmental and Ecological Physiology of Animals*,” we provide a set of eight original research articles devoted to the understanding of how redox metabolism allows animals to adapt to environmental changes. These works were produced by researchers from Argentina, Brazil, Colombia, Egypt, Germany, Japan, Serbia, South Africa and Sweden.

Aquatic organisms are often used for biomonitoring purposes in ecotoxicology. The measurement of specific biomarkers, including redox biomarkers, allows the assessment of the effects of physical, chemical or biological agents on organisms. In this context, the work by Sayed et al. characterized morphological alterations and nuclear abnormalities in erythrocytes of medaka (*Oryzias latipes*) irradiated with low-dose gamma radiation. They also found the manifestation of these abnormalities depends on p53, a redox-sensitive transcription factor that controls antioxidant genes. In a study with another fish, Hamed et al. identified eryptosis and poikilocytosis in red blood cells of Nile tilapia (*Oreochromis niloticus*) as biomarkers of exposure to microplastics. The levels of abnormal erythrocytes and nuclear abnormalities remained altered even after 15 days of recovery, indicating long-term cytotoxic and genotoxic effects of microplastics on tilapia. One common limitation of ecotoxicology studies is to ignore the possible effects of feeding status and naturally occurring toxins on a given biomarker. Gorokhova and El-Shehawy tackled this issue by testing the effect of toxin-producing cyanobacteria in the diet of two copepods, *Acartia bifilosa* and *Eurytemora affinis*, on oxidative biomarkers, growth and reproduction. Their findings suggest that biochemical responses to feeding activity and diet should be accounted for when using biomarker profiles in field-collected animals. This can help refine terms for assessing biomarkers of environmental stress and metabolic mechanisms in aquatic animals that consume toxic cyanobacteria.

Seasonal changes in environmental water availability are critical climatic events in the life history of animals. For example, many species synchronize their breeding period with the environment so that their offspring can make use of abundant water and food resources; some species retreat into microhabitats, suppresses their metabolism and remain inactive, minimizing their need for resources as they estivate during dry seasons; and other species employ phenotypic plasticity mechanisms to accelerate development to achieve terrestrial forms earlier when faced with water restriction. Moreira et al. investigated how enzymatic antioxidants and glutathione in the skeletal muscle are affected by metabolic depression in free-ranging frogs (*Pleurodema diplolister* and *Proceratophrys cristiceps*) naturally estivating in the wild. For these two species of the Brazilian Caatinga, the dry season (i.e., estivation) was associated with higher activities of catalase and glutathione peroxidase. Although both species use the same microhabitat and survival strategy (i.e., to estivate) during the dry season, the study identified biochemical difference between them, which might be related to their differences in behavior and metabolism. In an attempt to shed light on the molecular mechanisms underlying the activation of endogenous antioxidants during estivation, Giraud-Billoud et al. focused on the biochemical responses of several tissues at the beginning of the estivation period of golden apple snail (*Pomacea canaliculata*). A general response observed for the three analyzed tissues (digestive gland, gill and lung) was an increase in oxidative damage to proteins and catalase activity after 7 days of estivation. Short-term estivation was also associated with the upregulation of FOXO, whereas other redox-sensitive transcription factors were downregulated or unresponsive. These findings highlight the possible role of FOXO in the regulation of the redox metabolism during estivation. Moreover, these studies further strengthen the Preparation for Oxidative Stress theory, originally proposed in the late 1990’s (Hermes-Lima et al., 1998), and later fully updated (Hermes-Lima et al., 2015). Prokić et al. set out to assess the carry-over effect, if any, of water availability during the larval stage on the response of yellow belly toads (*Bombina variegata*) to food deprivation once they had metamorphosized to juveniles. Similar to the aforementioned results for estivating snails and frogs, desiccation during the larval stage led to the upregulation of selected endogenous antioxidants. Thus, there was a carry-over effect on the redox metabolism in toads indeed. Fasting, on the other hand, elicited oxidative damage to lipids and was associated with downregulation of antioxidant enzymes and glutathione regardless of previous water regime during metamorphosis.

Lastly, a couple of studies investigated the interplay between reproductive investment and redox metabolism in the context of life history trade-offs. Jacobs et al. used oxidative stress markers to gauge the physiological cost of reproduction in highveld mole-rats (*Cryptomys hottentotus pretoriae*), a cooperatively breeding mammal. Using a non-lethal sample collection approach, the authors found a sex-dependent cost of reproduction associated with seasonal changes in the environment, physiology and behavior. Malod et al. tested the role of accumulated oxidative damage on the life history tradeoff between reproduction and longevity by comparing lines of flies selected for early oviposition and shorter lifespan. In this selection line, the tradeoff already happened, and the outcome was shorter lived flies with higher reproductive output. The authors expected to find high oxidative damage and reduced antioxidants defenses in these flies but instead found diet and housing (wild vs. lab) were connected to higher accumulate oxidative damage. Their data indicate that oxidative damage does not play a role after the tradeoff is established, but it is possible that oxidative damage had a role prior to the establishment of said tradeoff.
